# A school-based intervention improves physical fitness in Ecuadorian adolescents: a cluster-randomized controlled trial

**DOI:** 10.1186/s12966-014-0153-5

**Published:** 2014-12-10

**Authors:** Susana Andrade, Carl Lachat, Angelica Ochoa-Aviles, Roosmarijn Verstraeten, Lieven Huybregts, Dominique Roberfroid, Diana Andrade, John Van Camp, Rosendo Rojas, Silvana Donoso, Greet Cardon, Patrick Kolsteren

**Affiliations:** Food Nutrition and Health Program, Universidad de Cuenca, Avenida 12 de Abril s/n y Avenida Loja, EC010107 Cuenca, Ecuador; Department of Food Safety and Food Quality, Ghent University, Coupure links 653, 9000 Ghent, Belgium; Institute of Tropical Medicine, Nationalestraat 155, 2000 Antwerp, Belgium; Department of Movement and Sports Sciences, Ghent University, Watersportlaan 2, 9000 Ghent, Belgium

**Keywords:** Fitness, Sedentary, Physical activity, Adolescents, Randomized control trial

## Abstract

**Background:**

Effective lifestyle interventions are needed to prevent noncommunicable diseases in low- and middle-income countries. We analyzed the effects of a school-based health promotion intervention on physical fitness after 28 months and explored if the effect varied with important school characteristics. We also assessed effects on screen time, physical activity and BMI.

**Methods and results:**

We performed a cluster-randomized pair matched trial in schools in urban Ecuador. The intervention included an individual and environmental component tailored to the local context and resources. Primary outcomes were physical fitness (EUROFIT battery), screen time (questionnaires) and physical activity (accelerometers). Change in BMI was a secondary outcome. A total of 1440 grade 8 and 9 adolescents (intervention: n = 700, 48.6%) and 20 schools (intervention: n = 10, 50%) participated. Data of 1083 adolescents (intervention: n = 550, 50.8%) from 20 schools were analyzed.

The intervention increased vertical jump (mean effect 2.5 cm; 95% CI 0.8-4.2; P = 0.01). Marginally insignificant, adolescents from the intervention group needed less time for speed shuttle run (intervention effect = −0.8 s, 95% CI −1.58-0.07; P = 0.05). The proportion of students achieving over 60 minutes of moderate-to-vigorous physical activity/day decreased over time with the change in proportion significantly less in the intervention schools (6 vs. 18 percentage points, P < 0.01). The intervention effect on speed shuttle run was significant in larger schools while the effect on vertical jump was larger in mixed gender school compared to small and female schools. The proportion of schools that met the recommendations for physical activity increased with 37% in intervention schools with half-day schedule compared to the controls in the pair. No significant effects were found on screen time and BMI. Measurement of physical activity in a subsample was a limitation. No adverse effects were reported.

**Conclusions:**

A school-based intervention with an individual and environment component can improve physical fitness and can minimize the decline in physical activity levels from childhood into adolescence in urban Ecuador.

**Trial registration:**

Clinicaltrials.gov identifier NCT01004367.

## Introduction

Lifestyle related noncommunicable diseases (NCDs) are a leading cause of death in low- and middle-income countries (LMICs) [1]. This is unfortunate, as they could be prevented by tackling risk factors such as physical inactivity [2], sedentary behavior (i.e. increased screen time) [3] and poor physical fitness [4]. It has been estimated that 1.3 million deaths worldwide can be prevented if the current physical activity recommendations were met [5]. Only a few LMICs have developed strategies to improve physical activity [6]. Adolescence is an important period for the prevention of NCDs [7] as physical inactivity [8] and poor physical fitness [9] at early adolescence are associated with the development of NCDs during adulthood. Furthermore, unhealthy lifestyles [10] and physical activity patterns [11] consolidate during adolescence and persist during adulthood. Schools are hence appropriate settings for health promotion programs [12,13]. Current evidence indicates that the most effective school-based interventions to increase physical activity [13] or tackle obesity [12] are those that involve individual and physical environment component [12,13]. However, evidence to prevent NCDs through school-based interventions in LMICs is of low quality [14,15]. The few school-based interventions from LMICs that aimed to promote an active lifestyle had important methodological limitations such as the absence of a theoretical framework to guide the interventions, a weak study design, or a lack of objectively measured outcomes [14]. This is worrisome as adequate evidence is needed to guide allocation of scarce resources to tackle NCDs in LMICs [16].

The results of a previous study among 12–15 year old adolescents in Cuenca, Ecuador showed that the prevalence of overweight/obesity was around 20% [17] and that 59% of adolescents had inadequate physical fitness levels [18]. We implemented a school-based health promotion intervention “ACTIVITAL” that aimed at improving diet and physical activity. ACTIVITAL was developed using participatory approaches and tailored to the Ecuadorian school context. In the present paper, we present the effectiveness of the trial on one set of the primary outcomes, i.e. physical fitness, screen time, physical activity and the effect on body mass index (BMI) as secondary outcome. In addition, we analyzed if the effect of the intervention varied with important school characteristics i.e. size, type, class schedule, gender composition and space for physical activity. Other primary outcomes (dietary intake and factors influencing dietary and physical activity behavior) are presented elsewhere to ensure sufficient detail in presenting the findings of the trial on physical fitness, activity and sedentary lifestyle outcomes.

## Methods

ACTIVITAL was a pair-matched cluster randomized controlled trial conducted from 2009 to 2012 in Cuenca, the third largest city in Ecuador and located at an altitude of ±2400 m. Cluster (school) randomization was chosen as the trial used a school-based approach. We report our findings according to the CONSORT guidelines [19]. This study was approved by two ethics committees, the “Comité de Biomedicina de la Universidad Central del Ecuador” from Quito - Ecuador (CBM/cobi-001 - 2008/462) and the Ghent University Hospital - Belgium (FWA00002482).

### Participants, sampling, allocation and blinding

Schools were eligible if: (i) they had >90 students in 8^th^ and 9^th^ grade, and (ii) they were located in the urban area of Cuenca, Ecuador. The eligible schools were paired according to four criteria: (i) total number of students of the school, (ii) monthly school fee (as proxy for the socio-economic status of the school), (iii) gender (male/female only or mixed gender) and (iv) time schedule of classes (morning: 7:00 to 13:00 or afternoon: 12:00 to 18:00). In Ecuador, large schools might divide the students in two groups because of logistic constraints. In this case the youngest students attend classes in the afternoon and the oldest students in the morning. Schools with no matching pair were excluded. Sample size was calculated on a nutritional outcome. Ten pairs and a sample size of 65 children per school was required to detect a reduction of 40% to 30% energy from fat using a two side significance level α = 0.05, variation in clusters means K_m_ = 0.15 and a power of 80% [20]. We assessed if the trial was sufficiently powered for each of the outcomes analyzed. All outcomes had a power >80% except for bent arm hang and 20 m shuttle run (65% and 64% respectively). The power calculations were obtained using the formula for sample size calculations of pair matched trials [20].

**Figure 1:**
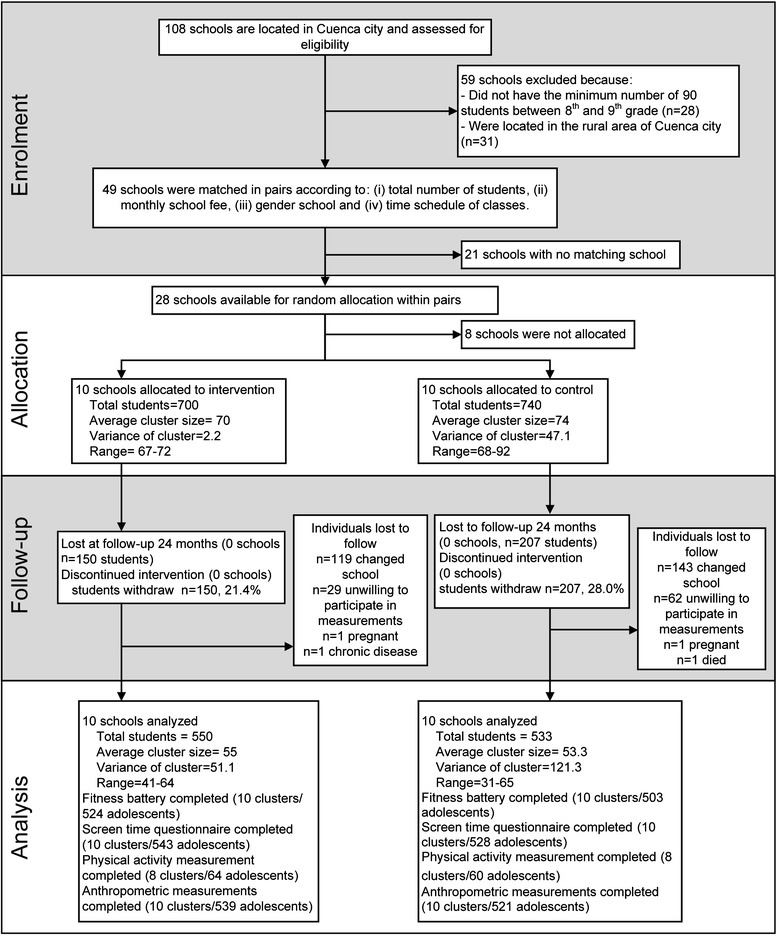
**Enrolment, allocation, follow-up and analysis of Ecuadorian adolescents in a school-based health promotion intervention.**

A total of 28 out of 108 schools were paired (Figure 1). We randomly selected 10 pairs in Stata (version 12.0, Stata Corporation, Texas, USA) using a random number generation with random allocation of the intervention within each pair. In each school, two 8^th^ grades and two 9^th^ grades were randomly selected and all students in those grades were invited to participate in the study. Pregnant adolescents and those with muscle or bone injuries or a concomitant disease were excluded at any time during the trial. In total we enrolled 1430 adolescents, including 10% of possible dropouts. After the pairs were selected and the school principal accepted to participate in the study (participation rate 100%) we obtained informed assent from adolescents (acceptance rate 85%) and written consent from caretakers (participation rate 90%). Adolescents were not informed about the existence of a counterfactual school.

### Interventions

The intervention program was developed using the results of needs assessment jointly with the intervention mapping (IM) protocol [21] and Comprehensive Participatory Planning and Evaluation (CPPE) [22]. A needs assessment was performed through qualitative (focus groups) and quantitative studies (physical fitness assessment). Results of focus groups with adolescents, parents and school staff showed that poor knowledge on physical activity and its importance, a preference for sedentary activities, laziness, time constraints, parent and peer support, and role modeling of famous Ecuadorian athletes were key individual and environmental determinants of physical activity in the study population [23]. The quantitative study (assessed using EUROFIT battery) showed that three out of five adolescents had a poor physical fitness [24].

Once the needs were identified, the CPPE approach [22] and the IM [21] were used to define intervention objectives. Using the inputs of the needs assessment and the IM [21], the exact behavior expected from adolescents was identified for each intervention objective. The most important and modifiable factors to reach each intervention objective were selected from the local evidence collected during the needs assessment, input received from the CPPE and existing literature. Afterwards, by crossing the expected behavior and modifiable factors, specified change objectives were developed to guide what adolescent or environmental agents needed to do to reach the intervention objectives. Next, behavioral change techniques that have been reported as effective [21,25] were identified and mapped against each factors. Finally, the selected behavioral change techniques (the social cognitive theory, information-motivation behavioral skills model, control theory, trans- theoretical model and theory of planned behavior.) were used together with input from CPPE to create strategies at individual and environmental level.

For physical activity specifically, the intervention objectives were: i) adolescents decrease daily screen time (1–2 hours/day), ii) adolescents increase daily physical activity levels to reach 60 min/day, and iii) the school offers more opportunities for being active. The individual strategy included the delivery of educational package organized at classroom level (Table 1). The persons in charge of delivering the educational package received an introduction to the intervention objectives and a basic workshop on healthy eating and physical activity. The physical activity environmental strategy included (i) workshops with parents that were organized at the same time as classes with adolescents and covering similar topics; (ii) organization of social events at school such as an interactive session with famous young athletes and (iii) environmental modification: a walking trail was drawn on the school playground in the second year of the intervention. There was no minimum dose for the activities for each of the intervention strategies. Details of the physical activity intervention are provided in Table 1. The control schools received the standard curriculum as determined by the Ecuadorian government. The latter is geared at increasing sports skills and schedules a mandatory 80 min of physical education per week.

**Table 1:** **Physical activity intervention components of the ACTIVITAL study implemented among 12–15 year old adolescents in 10 schools of Cuenca – Ecuador during 2010–2012: description of strategies, dose and response** ^*^

**What**	**Who/where**	**Why**	**When**	**How**	**Dose/response**
**1. Individual-based strategies**					
**Book 1 (Curriculum)**	School teachers and trained staff/ classroom	- To create awareness regarding the importance of an adequate physical activity throughout adolescence	September 2010 - February 2011	Thought textbooks and pedagogic materials for teachers and students. The material contained educational objectives, clear instructions for implementation the physical and educational activities during the classes without additional training.	Dose: 100% of classes addressing physical activity component were delivered
One out of five chapters addressed physical activity and sedentary behavior. This chapter was developed to be delivered in 90 minutes					
			Each chapter was performed every two weeks.		
					Response: The students had a 95% of average attendance of classes on physical activity around 75% of adolescents showed an active participation in the classes 54% of the scheduled classes addressing physical activity component were delivered by the school teacher
		- To increase knowledge and enhance decision-making skills			
**Book 2 (Curriculum)**	School teachers and trained staff/classroom	- To encourage the adolescents to be physically active for at least 60 min per day and to spend maximum 2 hour per day on sedentary activities.	September 2011-January 2012. Each chapter was performed every two weeks.	A second set of textbooks and pedagogic materials were developed for teachers and students. The material contained educational objectives and clear instructions for implementing the physical and educational activities.	
The book contained 8 chapters in total and one corresponded to the physical activity. Chapter 7: Physical Activity (how to remove barriers in order to be more physically active). This chapter was planned to be delivered in 90 minutes.					
**2. Environment-based strategies**					
**Parental workshops**	ACTIVITAL staff/school meeting room	- To support healthy behavior of adolescents at home - To increase the awareness of parents regarding the importance of regular physical activity for adolescents, how to be active during the day and how to deal with barriers to be physically active.	1 workshop from October 2010 till February 2011 1 workshop from October 2011 till January 2012	Workshops of 1 hour were delivered by the ACTIVITAL staff. Parents attendance was mandatory through a letter signed by each school principal Each leaflet included theoretical information, advises and benefits on the particular topic of the workshops	Dose: Two workshops (100%) related to physical activity component were delivered as planned. Response: Around 10% of the parents attended both workshops. Around 97% of the parents showed an interest in the contents of the workshops
In total six workshops were performed. Informative leaflets supporting the content of the workshop were distributed to each participant during the workshops. Two workshops focused on decreasing sedentary time and increasing physical activity (1^st^ year) and dealing with barriers for physical activity (2^th^ year).					
**Social event**	Young athletes/auditorium	To encourage physical activity through the positive influence of social models	Once during the intervention	A 1-hour interactive session with young athletes was given. Athletes shared their personal sport experiences and gave advice on active lifestyles and physical activity.	Dose: One pep talk was delivered in each school (100%)
-Pep talks by successful and well-known young male (n = 3) and female (n = 2) athletes, which were international young champions in BMX, swimming, racquetball and weightlifting					
					Response: Around 78% of adolescents showed an interest in the pep talks.
**Walking trail and posters**	Physical education teacher/classroom	- To increase availability and accessibility to opportunities for physical activity inside the schools	September 2011 – January 2012	The physical education teacher explained the students about the importance of being physically active and how the students could use the walking trail to be more active during recess.	Dose: The walking trail was implemented in the ten schools (100%)
- Using line markings, a walking trail was drawn on the school’s playground. The length of the trail was the perimeter of playground.					
					Response: Around 25% of the adolescents used the walking trail according to the results of the two schools where the walking trail was evaluated.
		- To motivate the students to walk more during the recess time			
- 3 posters suspended on the school walls adjacent to the trail, with phrases like: *“Do you like to talk? Walk and Talk*”					
**Posters for classroom and food tuck shop**	ACTIVITAL staff/classroom and food tuck shop	- To encourage students to be active and eat healthy	Monthly from October 2010 to February 2011	Posters included key messages to be active were suspended on the classroom walls and in front of the food tuck shops.	Dose: The five posters (100%) were suspended in the classroom and food tuck shop
Fiver different posters with key messages on physical activity and pictures of the young athletes					

^*^The “ACTIVITAL” trial aimed at improving diet and physical activity. This table summarizes the physical activity component of the trial, which aimed at improving both physical activity and sedentary behavior.

ACTIVITAL started in October 2009 and finished in June 2012 i.e. through the academic years 2010–2012 with a total duration of 28 months. Once started it was only interrupted by annual break (July and August). In the first academic year we started with the baseline measurements (October 2009 - February 2010) and the application of the IM protocol and CPPE (March-June 2010). In the second year we implemented a first part of the intervention (September 2010 - February 2011) and performed an intermediate follow-up (March-June 2011) in which physical fitness and physical activity was not measured. In the third year, the second part of the intervention was implemented (September 2011- January 2012) and final measurements were performed (from February 2012 - June 2012).

To assess progress, monitor potential adverse effects and coordinate the intervention activities, research staff met with schoolteachers and school managements every two to three weeks. One person in each school (mostly the medical doctor or the school supervisor) was assigned as contact point between research staff and the schools.

### Outcome measures

The measurements were performed when students entered the 8^th^ and 9^th^ grade (12.3 and 13.3 years respectively) and after 28 months, at the end of the academic year. Medical doctors, nutritionists and health professionals with field experience received a 40-hour training session to assess outcomes. Reliability and repeatability were assessed to assure precision and accuracy of the measurements. The research team provided regular supervision.

#### Physical fitness

The EUROFIT test battery was used to assess physical fitness in different dimensions with nine tests [26]: cardio-respiratory endurance (20 m shuttle run test), strength (handgrip and vertical jump test), muscular endurance (bent arm hang and sit-ups test), speed (speed shuttle run and plate tapping), flexibility (sit-and-reach) and balance (flamingo balance test). This battery was previously validated in adolescents [27,28] and applied in various Latin American countries [29-32] to assess physical fitness. The vertical jump is a variation of the original item of EUROFIT (standing broad jump) and is valid for the assessment of muscular strength [33]. Physical fitness data were used as continuous variables. An increase in test results indicated higher physical fitness, apart from the speed shuttle run, the plate tapping and flamingo balance tests for which lower scores indicated better fitness.

#### Screen time

Screen time was assessed using a validated self-reported questionnaire [34]. Adolescents reported the number of hours per typical week and weekend day that they spent watching television, playing videogames or using the computer. Response categories were “zero”, “30 min”, “1 hour”, “2 hours”, “3 hours”, “4 hours”, “5 hours” and “> 6 hours”. Screen time was treated as a continuous variable. We also assessed the proportion of adolescents with screen time >3 hours/day at the end of the trial as this cut-off has been shown to be associated with a higher likelihood of metabolic syndrome [34].

#### Physical activity and sedentary time

Physical activity and sedentary time were assessed using accelerometers (type GT-256 and GT1M Actigraph, Florida USA). Due to the high cost of the accelerometers, we assessed physical activity in a subsample of adolescents that was selected using a random number in Stata. The MAH/UFFE Analyzer (version 1.9.0.3) and a syntax in Stata were used for data reduction and to compute registered time, the time spend on sedentary (≤100 counts/min), light (100–759 counts/min) and moderate to vigorous physical activity (≥760 counts/min) [35]. Accelerometers were worn for 5 weekdays. As recommended, the first and last day of measurement were excluded from the analyses as well as those registrations with less than 540 min of registered time per day [35]. Accelerometer data were adjusted for the total registered time. The proportion of adolescents who met the recommended 60 min [36] of moderate to vigorous physical activity per day was calculated.

#### BMI

Weight was measured using a digital calibrated balance (SECA 803, Hamburg, Germany) and recorded to the nearest 100 g. Height was recorded to the nearest mm by using a mechanical stadiometer (PORTROD, Health o Meter, Illinois, USA). Students were measured with light clothing and without shoes in a separate room by a researcher of the same gender. All anthropometric measurements were done twice and average values were used. BMI indices were calculated using Anthro plus (version 3.2.2, WHO Geneva, Switzerland) and established cut-offs [37]. BMI z-score was used as an outcome. We also assessed the effect of the intervention on the proportion of adolescents with a BMI in the healthy range [37] at the end of the study.

### Other measurements

The adolescent’s knowledge on recommendations and the importance of physical activity in adolescent health was assessed using a questionnaire before the classes.

The socio-economic status of the adolescent’s household was defined according to the Integrated Social Indicator System for Ecuador [38]. The system classifies a household as “poor” when they report to have no access to education, health, nutrition, housing, urban services or employment, otherwise the household is classified as “better-off”.

The following school characteristics were measured prior to the intervention: (i) school size as binary variable (0 = small schools; 1 = large schools) with the median (n = 695) of the school size as cut-off; (ii) type of school as a binary variable (0 = public; 1 = private); (iii) school schedule as a binary variable (0 = half day; 1 = full day schedule); (iv) school gender as a binary variable (0 = both genders; 1 = female only). The sample did not contain schools with only male students; and (v) physical activity space, expressed number of students/m^2^ of space available for being physically active in each school. The median (4.07 students/m^2^) was used as a cut-off for this.

Using a pre-defined process evaluation framework and instruments, we also monitored the delivery of the intervention. Researchers recorded attendance and participation rates during classes and the receptiveness of the adolescents to the classes. Teachers in charge of a class filled out a questionnaire at the end of each class to assess their appreciation of the materials and the messages conveyed. We assessed if adolescents noticed, liked and used the walking trail using a questionnaire in a convenience sample of 2 schools. At the end of the workshop with parents, a questionnaire was administered to parents to measure satisfaction and to get general feedback of the workshops. In the present manuscript we describe the delivery and response of the intervention. A full process evaluation will be reported separately.

### Statistical analysis

Differences at baseline between intervention groups as well as differences dropout and remainders groups were assessed using a t-test for continuous variables adjusted for the pair matched allocation and χ^2^ test for categorical variables. To estimate the effect of the intervention, we used a difference in differences approach.

An intention-to-treat analysis was performed to assess the intervention effect using mixed linear regression models with the pair-matching as random effect. In such models, the Beta coefficient of the intervention variable indicates the difference in means for continuous dependent variables and the difference in absolute risks for dichotomic ones [39]. All models were adjusted for baseline BMI z-score, gender, adolescent socio-economic status and knowledge on recommendations and health benefits of physical activity. We adjusted the analysis for prior knowledge on physical activity as it influences on how much the new knowledge can be assimilated [40]. Akaike-Schwartz criteria [41] were used to determine the optimal covariance structure. To assess the effect of the adjusting, we also analyzed the effect of the intervention using crude models. We also tested variations of the effect by pairs of schools by a meta-analysis with visual appraisal of the forest plot and heterogeneity statistics (I^2^) [42].

Next, we tested if the intervention had a different effect in boys and girls using interaction term gender × allocation group. As there was substantial heterogeneity among the pairs we explored if the intervention effect was modified by school characteristics for outcomes with a P < 0.1 in the main analysis. For each outcome, we first assessed the effect modification for the five school characteristics in separate models (bivariate models) by including the interaction term of the school characteristic × intervention. Secondly, a final model was constructed with all school characteristics that were significantly (P < 0.05) associated with the outcome in the bivariate models. Finally, the analysis was stratified when the interaction terms were significant (P _interaction_ < 0.1). In addition, we tested the effect of missing data for all outcomes with P < 0.1 using a multiple imputation method based on chained equations with 50 imputation runs. Age, BMI z-score, gender, physical activity knowledge and socio-economic status were used as predictors in models to impute data in the pairs. All tests were performed with a significance level of 5% and models were evaluated for collinearity using variance inflation factors. Given the small number of pairs (n = 10), we calculated the P*-*values from the multilevel analyses from a t-distribution with 9 degrees of freedom. Data were analyzed using Stata.

## Results

A total of 1440 adolescents (intervention group: n = 700, 48.6%) and 20 schools (intervention group: n = 10, 50%) participated in the trial (Figure 1). Baseline characteristics at individual and cluster level were comparable (Tables 2 and 3). Except for 20 m shuttle run, handgrip, plate tapping, sedentary time and light physical activity time, there were no significant differences in baseline characteristics.

**Table 2:** **Participant characteristics at baseline**

	**n** ^**a**^	**Intervention group**	**Control group**
		**Mean (SD** ^**b**^ **)**	**Mean (SD** ^**b**^ **)**
Age	1378	12.9 (0.8)	12.9 (0.8)
Female (%)	1440	66.4	59.3
**Fitness**			
*Cardiopulmonary fitness*			
20 m shuttle run (stage)	1363	2.5 (0.7)	2.7 (0.9)
20 m shuttle run (min)	1362	1.7 (0.7)	1.9 (0.9)
*Speed-agility*			
Speed shuttle run (s)	1389	24.6 (2.4)	24.5 (2.2)
Plate tapping (s)	1394	14.6 (1.9)	14.2 (2.0)
*Flexibility*			
Sit and reach (cm)	1391	20.1 (6.6)	20.5 (6.3)
*Muscle strength and endurance*			
Sit-up (number/30 s)	1389	12.0 (3.9)	12.6 (3.4)
Vertical jump (cm)	1391	25.4 (5.6)	26.0 (5.3)
Bent arm hang (s)	1390	6.0 (7.0)	6.0 (6.6)
Handgrip (kgf)	1393	18.4 (4.9)	19.2 (5.0)
*Balance*			
Flamingo (trying/min)	1389	17.8 (5.8)	18.5 (6.0)
**Screen time**		Median (IQR)	Median (IQR)
TV in the week (h/day)	1370	1.0 (0.5 – 2.0)	1.0 (0.5 – 2.0)
TV in the weekend (h/day)	1370	2.0 (1.0 – 4.0)	2.0 (1.0 – 4.0)
Internet in the week (h/day)	1370	0.5 (0.0 – 1.0)	0.5 (0.0 – 1.0)
Internet in the weekend (h/day)	1370	0.5 (0.0 - 1.0)	0.5 (0.0 – 1.0)
Video games in the week (h/day)	1370	0.0 (0.0 – 0.0)	0.0 (0.0 – 0.0)
Video games in the weekend (h/day)	1370	0.0 (0.0 – 1.0)	0.0 (0.0 – 1.0)
Total screen time in the week (h/day)	1370	2.5 (1.5 – 3.5)	2.0 (1.5 – 3.5)
Total screen time in the weekend (h/day)	1370	4.0 (2.0 – 5.5)	4.0 (2.0 – 6.0)
%Sedentary week (% screen time >3 h/day)^d^	1370	31.2 (46.4)	28.9 (45.4)
%Sedentary weekend (% screen time >3 h/day)^d^	1370	50.9 (50.0)	50.2 (50.0)
**Accelerometer data**		Mean (SD)	Mean (SD)
Total PA (counts/day)	226	305226 (109950)	280819 (116144)
Total PA (CPM/day)	226	375.5 (138.0)	357.8 (141.8)
Sedentary time (min/day)	226	487.6 (126.9)	484.9 (108.3)
Light PA (min/day)	226	217.6 (58.1)	192.7 (66.7)
Moderate-Vigorous PA (min/day)	226	119.3 (42.4)	109.4 (45.0)
% who meet the PA recommendation (60 min MVPA/day)	226	95.0 (21.9)	91.5 (28.0)
**Anthropometry**			
Body mass index (kg/m^2)	1382	19.8 (3.4)	19.7 (2.9)
Body mass index z-score	1371	0.3 (1.1)	0.3 (1.0)
Overweight prevalence (%)^c^	1371	20.1 (40.7)	19.7 (39.8)

^a^Total number of students.

^b^Adjusted for clustering.

^c^Overweight and obese combined.

^d^Based on the recommended 3 h per day maximum of screen time for adolescents.

CPM: counts per minute; MVPA: moderate to vigorous physical activity; PA: physical activity; SD: standard deviation.

**Table 3:** **School characteristics at baseline**

	**n** ^**a**^	**Intervention group**	**Control group**
		**Mean (SD)**	**Mean (SD)**
Age	20	12.8 (0.2)	12.9 (0.3)
Female (%)	20	66.1	57.6
**Physical fitness**			
*Cardiopulmonary fitness*			
20 m shuttle run (stage)	20	2.5 (0.2)	2.7 (0.3)
20 m shuttle run (min)	20	1.7 (0.2)	1.9 (0.4)
*Speed-agility*			
Speed shuttle run (s)	20	24.6 (11.2)	24.5 (6.6)
Plate tapping (s)	20	14.6 (2.6)	14.2 (5.9)
*Flexibility*			
Sit and reach (cm)	20	20.1 (1.4)	20.5 (1.2)
*Muscle strength and endurance*			
Sit-up (number/30s)	20	12.0 (0.9)	12.6 (0.8)
Vertical jump (cm)	20	25.4 (1.5)	26.1 (1.1)
Bent arm hang (s)	20	6.0 (23.1)	6.1 (19.6)
Handgrip (kgf)	20	18.4 (0.7)	19.3 (0.8)
*Balance*			
Flamingo (trying/min)	20	17.9 (1.3)	18.5 (1.4)
**Screen time**		Median (IQR)	Median (IQR)
TV hours in the week (h/day)	20	1.0 (1.0 – 1.5)	1.3 (1.0 – 1.5)
TV hours in the weekend (h/day)	20	2.0 (2.0 -3.0)	2.0 (2.0 -2.0)
Internet hours in the week (h/day)	20	1.0 (0.5 -1.0)	0.5 (0.4 -1.0)
Internet hours in the weekend (h/day)	20	0.5 (0.0 -1.0)	0.3 (0.0 -1.0)
Video games in the week (h/day)	20	0.0 (0–0)	0.0 (0–0 )
Video games in the weekend (h/day)	20	0.0 (0.0 – 0.1)	0.0 (0.0 – 0.1)
Total screen time week (h/day)	20	2.3 (2.0 – 2.5)	2.0 (1.9 – 2.3)
Total screen time weekend (h/day)	20	3.5 (3.0 – 4.0)	3.5 (3.0 – 4.1)
% Sedentary week (% screen time >3 h/day)	20	31.2 (11.1)	28.9 (9.6)
% Sedentary weekend (% screen time >3 h/day)	20	50.9 (8.6)	50.2 (9.4)
**Accelerometer data**		Mean (SD)	Mean (SD)
Total PA (counts/day)	18	308630.8 (38106.2)	274071.9 (46469.4)
Total PA (CPM/day)	18	381.4 (68.6)	348.4 (71.4)
Sedentary time (min/day)	18	480.0 (78.7)	492.8 (46.4)
Light PA (min/day)	18	223.6 (21.1)	191.1 (34.5)
Moderate-vigorous PA (min/day)	18	122.7 (21.0)	106.7 (16.2)
% who meet the PA recommendation (% >60 min MVPA/day)	18	95.0 (6.9)	93.6 (7.1)
**Anthropometry**			
Body mass index (kg/m^2)	20	19.8 (0.5)	19.7 (0.4)
Body mass index Z-score	20	0.3 (0.2)	0.3 (0.1)
Overweight prevalence (%)^b^	20	20.8 (7.5)	19.6 (3.6)

^a^Total number of clusters.

^b^Overweight and obese combined.

CPM: counts per minute; IQR: interquartile range; MVPA: moderate to vigorous physical activity; PA: physical activity; SD: standard deviation.

All schools completed the trial and the sample size included in the analyses was 1083 adolescents (63.2% girls, intervention group: n = 550, 50.8%). The attrition rate was higher (P < 0.001) in the control (28%, n = 207/740) compared to the intervention group (21%, n = 150/700). Most of the attrition was due to adolescents changing schools (73%, 262/357). Physical activity could not be assessed in 47% (117/251) of the adolescents as accelerometers malfunctioned (n = 70) or participants were lost to follow-up (n = 39 left school). Six students declined to participate and 2 were pregnant. In one school, no accelerometer readings were available for the same reasons. Adolescents lost to follow-up had a higher baseline score in the handgrip (P < 0.001) compared to adolescents who completed the trial, while the speed shuttle run (P = 0.01) and screen time during the week (P = 0.01) were better among the adolescents who completed the trial. There was no difference for all other outcomes. At the end of the intervention adolescents were on average 15.1 year ± 0.7.

### Primary outcomes

Adolescents from the intervention group had a greater increase in vertical jump (intervention effect = 2.5 cm; 95%CI 0.78-4.23; P = 0.01). Adolescents from the control group needed more time for speed shuttle run but this difference was small with borderline statistical significance (intervention effect = −0.8 s, CI-1.58-0.07; P = 0.05) compared to the intervention group indicating a deterioration of physical fitness for this component. Adolescents from the control group needed less attempts to keep their balance for the duration of one minute in flamingo balance test (P = 0.02) compared to intervention group (Table 4) i.e. the control group had a higher improvement in balance test compared to intervention group.

**Table 4:** **Group differences and mean changes in fitness, screen time, physical activity and BMI after intervention**

**Outcomes**		**Differences at individual level**		**Unadjusted effects at individual level** ^**c**^		**Adjusted effects at individual level** ^**e**^				**Effects in the pairs** ^**g**^		
	**n** ^**a**^ **(cluster)**	**Δ I (SD)** ^**b**^	**Δ C (SD)** ^**b**^	**Beta**	**P** ^**d**^	**Beta**	**P** ^**f**^	**ICC**	**95% CI**	**Effect size** ^**h**^	**95% CI**	**I** ^**2**^
**Physical fitness**												
*Cardiopulmonary fitness*												
20 m shuttle run (min)	**1003 (20)**	−0.17(0.83)	−0.02(1.19)	−0.18	0.18	−0.19	0.16	0.15	[−0.54 – 0.16]	−0.19	[−0.52 – 0.14]	89.3
*Speed-agility*												
Speed shuttle run (s)^i^	1021 (20)	1.89 (2.09)	2.69 (3.44)	−0.72	0.06	−0.76	0.05	0.15	[−1.58 - 0.07]	−0.81	[−1.67 - 0.04]	83.8
Plate tapping (s)^i^	1043 (20)	−0.18 (2.39)	0.36 (2.64)	−0.61	0.13	−0.70	0.10	0.32	[−1.70 - 0.31]	−0.64	[−1.56 - 0.34]	93.3
*Flexibility*												
Sit and reach (cm)	1040 (20)	1.84 (4.93)	1.97 (4.61)	−0.13	0.37	0.11	0.39	0.06	[−0.64 - 0.86]	−0.13	[−0.96 - 0.71]	52.7
*Muscle strength and endurance*												
Sit-up (number/30 s)	1031(20)	2.45 (3.81)	2.52 (4.15)	−0.04	0.47	0.15	0.36	0.11	[−0.63 - 0.92]	−0.002	[−0.95 - 0.94]	76.3
Vertical jump (cm)	1038 (20)	1.94 (6.80)	0.07 (6.45)	1.83	0.03	2.51	0.01	0.12	[0.78 - 4.23]	1.74	[0.12 - 3.36]	77.6
Bent arm hang (s)	1019 (20)	−0.64 (7.72)	0.005(7.38)	−0.70	0.27	−0.11	0.45	0.03	[−1.67 – 1.45]	−0.68	[−2.63 – 1.27]	82.8
Handgrip (kgf)	1032 (20)	5.86 (5.42)	5.70 (6.3)	−0.03	0.48	0.59	0.12	0.06	[−0.32 - 1.50]	0.076	[−1.49 - 1.34]	81.3
*Balance*												
Flamingo (trying/min)^i^	571 (20)	−1.69 (6.60)	−4.08 (7.60)	2.36	0.01	1.83	0.02	0.07	[0.25 - 3.41]	2.34	[0.53 - 4.14]	59.1
% able to do the flamingo test	1034 (20)	5.15	5.49	-0.05	0.08	−0.37	0.13	0.03	[−0.10 – 0.02]	−0.52	[−0.12 – 0.02]	38.3
**Screen time**												
Screen time in week day (h/day)	1071 (20)	2.02 (3.22)	1.83 (2.83)	0.20	0.17	0.29	0.11	0.01	[−0.15 - 0.73]	0.2	[−0.28 - 0.68]	48.1
Screen time in weekends (h/day)	1071 (20)	2.00 (3.66)	2.24 (3.85)	−0.23	0.27	−0.24	0.26	0	[−0.93 - 0.45]	−0.2	[−0.96 - 0.55]	64.2
% sedentary in week (screen time >3 h/day)	1071 (20)	2.57%	11.16%	−0.05	0.07	−0.06	0.06	0.01	[−0.12 - 0 .01]	−0.05	[−0.11 - 0.01]	0.0
% sedentary in weekends (screen time >3 h/day)	1071 (20)	−23.57%	−27.46%	0.05	0.07	0.04	0.08	0	[−0.10 - 0 .01]	0.05	[−0.03 - 0.13]	58.9
**Accelerometer data**												
Total PA (counts/day)	134 (18)	−17503 (143300)	−26291 (146553)	22356^j^	0.22	27804^j^	0.18	0	[−29525 - 85135]	37000	[−44000 - 120000]	61.3
Total PA (CPM/day)	134 (18)	−6.7 (156.2)	−15.3 (183.6)	18.8^j^	0.30	30.2^j^	0.23	0	[−46.2 -106.6]	47.8	[−42.5 - 138.1]	64.1
Sedentary time (min/day)	134 (18)	26.3 (149.6)	44.1 (158.9)	−14.4^j^	0.21	−18.1^j^	0.15	0	[−50.8 - 14.6]	−14.0	[−78.9 - 50.8]	42.8
Light PA (min/day)	134 (18)	−47.9 (69.2)	−47.1 (70.3)	4.3^j^	0.32	4.6^j^	0.32	0	[−14.6 - 23.8]	−6.1	[−42.8 - 30.6]	63.7
MVPA (min/day)	134 (18)	−8.8 (54.0)	−14.7 (55.1)	10.4^j^	0.17	13.6^j^	0.08	0	[−4.1- 30.8]	15.7	[−14.1 - 45.4]	59.1
% who meet the PA recommendation (60 min MVPA/day)	134 (18)	−5.87%	−18.09%	0.16	<0.01	0.20	<0.01	0	[0.07 - 0.33]	0.06^k^	[−0.41 - 0.53]^k^	58.1^k^
**Anthropometry**												
Body mass index (z-score)	1062 (20)	−0.09 (0.58)	−0.09 (0.52)	0.02	0.34	−0.01	0.38	0.02	[−0.09 - 0.06]	−0.004	[−0.09 - 0.08]	41.1
Overweight prevalence (%)^m^	1062 (20)	−0.56	−1.62	0.03	0.15	0.02	0.31	0	[−0.05 - 0.08]	0.03	[−0.03 - 0.09]	32.5

^a^Total number of students (clusters).

^b^Standard deviation adjusted for clustering.

^c^Crude models without covariates and adjusted for clustering.

^d^P-value for crude models.

^e^Adjusted for clustering.

^f^Multilevel random effect models adjusted for BMI z-score, gender, socio-economic status and the physical activity knowledge at baseline. P-values were calculated with t distribution with 9 degree of freedom.

^g^Random effect meta-analysis with pairs as random effect.

^h^Pooled unstandardized mean differences.

^i^Lower scores indicated better fitness.

^j^Models were adjusted for total time registered at baseline and at follow-up.

^k^The results were obtained from only two pairs. In all other pairs, at least one school had all students meeting the recommendation on MVPA.

^m^Overweight and obese combined.

Δ I: mean difference of the outcomes measured before and after the intervention in the intervention group; Δ C: mean difference of the outcomes measured before and after the intervention in the control group; CPM: counts per minute; ICC: intraclass correlation; I^2^: heterogeneity; PA: physical activity, MVPA: moderate to vigorous PA.

**Figure 2:**
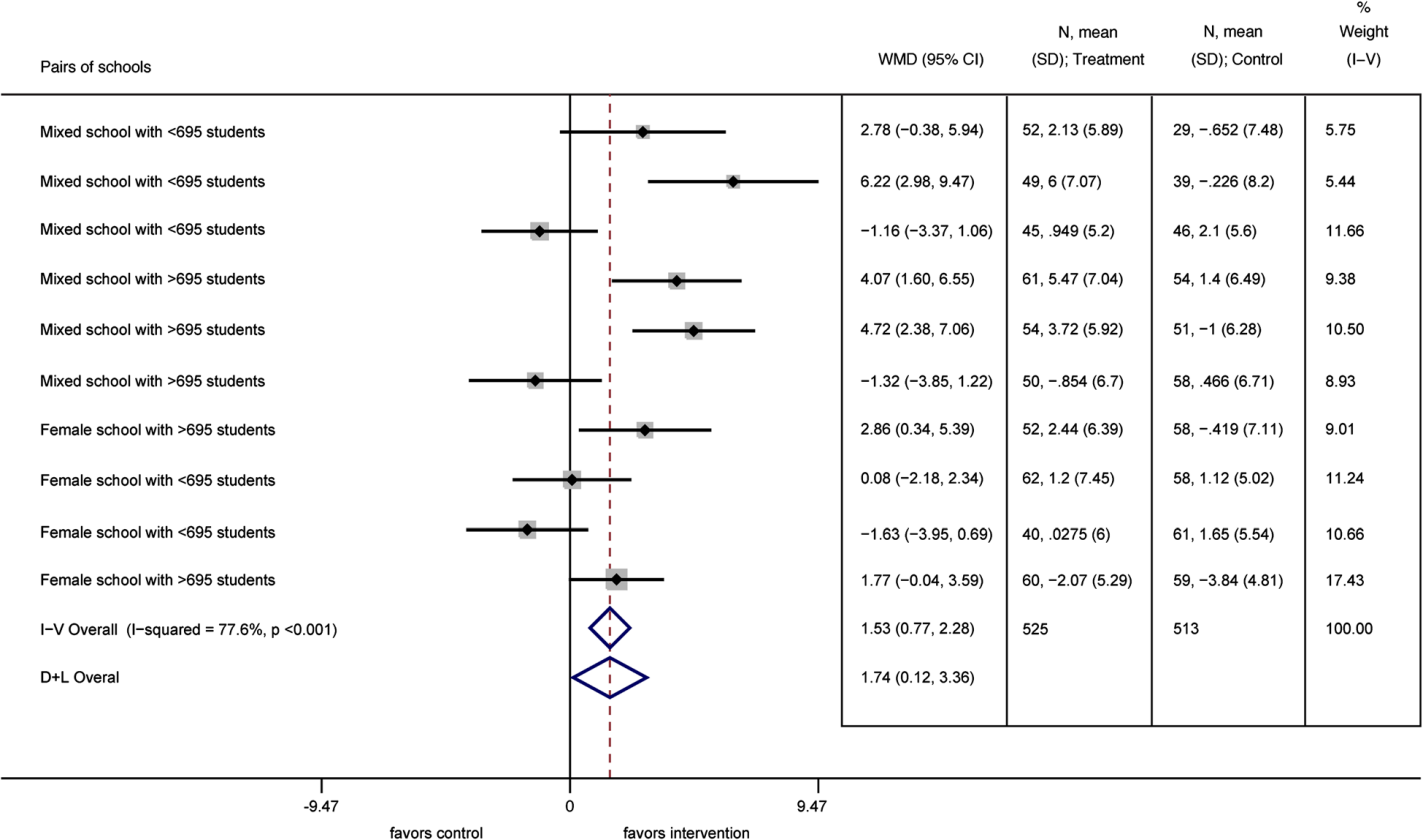
**Forest plot for vertical jump according to size and gender of the school pairs.**

Except for a few outcomes only a small fraction (<10%) of the intervention effect was explained by the pairs (intraclass correlation reported in Table 4). In addition, we observed a moderate to high (>25%) [42] heterogeneity in the intervention effect between pairs of schools for all but one outcome. Figure 2 illustrates this effect heterogeneity amongst pairs of schools for vertical jump (Table 4).

Over the intervention period, screen time increased around 2 hours in both intervention and control groups (Table 4). Marginally insignificant however, the proportion of adolescents with >3 hours of screen time during the week decreased with 6% in the intervention group. In the weekend days, this proportion increased with 4% respectively after the trial.

Total physical activity level decreased in the intervention group and control group and both groups had a similar increase in sedentary time (Table 4). At baseline, more than 90% of the adolescents had >60 min of moderate to vigorous physical activity per day. After the intervention, the proportion of adolescents that met this recommendation decreased significantly less in the intervention group compared to the control group (6 vs. 18 percentage points, P < 0.01).

### Secondary outcomes

The intervention did not lead to differences in changes of BMI z-score or prevalence of overweight.

### Ancillary analyses

Similar findings were obtained when analyzing the effect of the intervention with the crude models (Table 4). In addition, the intervention effect was not different among boys and girls.

School characteristics modified the intervention effect significantly for various outcomes (Table 5). In the full model the intervention effect on speed shuttle run was modified by school size (P _interaction_ < 0.01), the effect on vertical jump by school gender (P _interaction_ = 0.03), the effect on flamingo balance test by physical activity space (P _interaction_ = 0.04), the effect on the proportion of adolescents with >3 h of screen time during the weekend by type of school (P _interaction_ = 0.05) and the effect on the proportion of adolescents that met the recommendations for physical activity by school schedule (P _interaction_ = 0.01). After the stratification, the speed shuttle run only showed an improvement in pairs of larger schools. The speed shuttle run showed a decrease of 1.5 s (P < 0.01) in larger schools. The improvement in the vertical jump was only significant in pairs of schools with both male and female children. Vertical jump increased with about 3.6 cm (P < 0.01) in intervention schools vs. control schools in pairs of schools with both male and female students. In schools with a physical activity space ≤4.07 students/m^2^, control schools showed a significant (P = 0.01) improvement in the flamingo balance test. The proportion of adolescents with >3 h of screen time during the weekend increased in 13% in private schools allocated in the intervention group (P = 0.01). In pairs of schools that offer half-day class only, the proportion of adolescents that met the recommendations for physical activity increased with 37% in intervention schools. That proportion decreased with 29% in the intervention schools in pairs that provide full-day classes.

**Table 5:** **Subgroup analysis of physical fitness, screen time, physical activity according to school characteristics**

**Outcome**	**n**	**Control group**	**Intervention group**	**Separate model per school characteristic**		**Full model adjusted for school characteristics**	
		**Mean (SD)**	**Mean (SD)**	**Difference [95% CI]** ^**a**^	**P**	**Difference [95% CI]** ^**b**^	**P**
**Speed shuttle run 10×5 (s)**							
School size	1021				<0.01^c^		<0.01^c^
≤695 students	476	1.58 (3.59)	1.93 (2.47)	0.30 [−0.55-1.14]	0.25	0.22 [−0.36 -0.80]	0.24
>695 students	545	3.63 (3.02)	1.85 (2.32)	−1.83 [−2.34 to −1.32]	<0.01	−1.49 [−2.20 to −0.78]	<0.01
**Vertical jump (cm)**							
School gender	1038				0.03^c^		0.03^c^
Male and female students	698	0.29 (6.82)	2.93 (6.70)	3.55 [1.73-5.36]	<0.01	3.57 [1.76-5.38]	<0.01
Only female students	340	−0.34 (5.67)	−0.30 (6.48)	0.14 [−2.21-2.49]	0.45	0.06 [−2.16-2.28]	0.48
**Flamingo (trying/min)**							
Physical activity space	571				0.04^c^		0.04^c^
≤4.07 (students/m^2^)	237	−5.26 (7.87)	−0.82 (6.40)	3.29 [0.97 - 5.61]	0.01	3.29 [0.97 - 5.61]	0.01
>4.07 (students/m^2^)	334	−3.38 (7.38)	−2.43 (6.68)	0.91 [−0.99 - 2.81]	0.19	0.91 [−0.99 - 2.81]	0.19
**% sedentary in weekends (screen time >3 h/day)**							
School type	1071				0.03^c^		0.05^c^
Private	438	0.18 (0.39)	0.31 (0.46)	0.13 [0.04 - 0.21]	<0.01	0.13 [0.03 - 0.23]	0.01
Public	633	0.26 (0.44)	0.25 (0.43)	−0.02 [−0.10 - 0.05]	0.29	0.00 [−0.14 - 0.13]	0.48
**Proportion of adolescents who meet the recommendation (60 min of MVPA/day)**							
School schedule	134				0.01^c^		0.02^c^
Half-day class	110	0.70 (0.46)	0.96 (0.19)	0.28 [0.14-0.42]	<0.01	0.37 [0.14 - 0.61]	0.01
Full-day class	24	1.00 (0.00)	0.77 (0.44)	−0.29 [−0.54 to −0.04]	0.02	−0.29 [−0.54 to −0.04]	0.02

^a^Differences and CI were obtained from a linear mixed model adjusted for BMI z-scores, gender, socio-economic status and physical activity knowledge at baseline, including the interaction term between school characteristic and allocation group.

^b^Differences and CI were obtained from linear mixed models adjusted for BMI z-scores, gender, socio-economic status and physical activity knowledge, including the interaction term between school characteristic and allocation group. Models included all school characteristics as fixed effects that were significantly associated with the outcome (P < 0.05) and all significant interaction terms (P < 0.1) from the separate models.

^c^P for interaction (significant at P < 0.10).

CI: confidence interval; SD: standard deviation.

The findings were similar after imputing missing variables and produced following estimates: vertical jump: β = 2.49, P = 0.01 (0.4% difference), speed shuttle run: β = −0.72, P = 0.07 (5.5% difference), flamingo balance test β = 2.26, P = 0.002 (24% difference), mean moderate to vigorous physical activity time: β = 12.0, P = 0.11 (11.6% difference) and the proportion meeting the recommended 60 min/day of moderate to vigorous physical activity β = 0.12, P = 0.05 (39% difference).

In the classes aimed to improve the physical activity there was large attendance and active participation (95% and 77% respectively). All classes were delivered and teachers reported that 95% of content was explained as scheduled. Most (79%) of the adolescents reported this to be new knowledge. The larger majority (85%) of adolescents that attended the classes, scored the quality of classes with >8/10. Almost all (99%) adolescents attended the sessions with athletes and 78% were considered attentive. The walking trails were drawn in all schools. The majority of the adolescents had noticed the walking trail and liked it (91% and 60% respectively) but only 25% reported using it during breaks. Amongst the parents (10% of all parents) that attended the workshops, 90% scored the quality of the workshop high (≥80%) and most (76%) considered this to be new information. No harm or adverse effects were reported by adolescents, teachers or school management staff in the control or intervention group during the trial.

## Discussion

We report how a school-based intervention had a positive effect on physical fitness parameters and recommendations for moderate to vigorous activity of adolescents in an urban area of Ecuador. The increase in muscular strength as measured by vertical jump corresponds to 10% of the average score at baseline in the intervention group. Ortega *et al*. have shown that higher muscular strength during adolescence is associated with better cardiovascular and skeletal health at adulthood [9]. The intervention also resulted in an improvement of the speed shuttle run corresponding to a relative time decrease of 3% compared to the baseline values. Albeit marginally insignificant, this effect is considerable and compares to differences in speed-agility between non-obese and obese adolescents [43]. The effect on the balance component of physical fitness was in favor of the control group. We attribute this counterintuitive finding to the fact that our intervention promoted physical activity and did not include specialized training for static activities needed for the balance test [44]. We also observed that adolescents in the control group engaged more in static games during breaks like hacky sack and throwing coins near a target, while those in the intervention group were encouraged to engage in sports and use the walking trail.

To our knowledge, trials in our age group from high-income countries have generally resulted in mixed effect on physical fitness [45-48]. Only one study in African American girls with high blood pressure led to a 1 min increase in step-test compared to children who only received physical education [49]. These results are less positive than those reported in the present study. This is surprising, as we did not provide an extra hour of physical education per week, exercises during the recess or specific equipment for physical activity. Instead, we developed a comprehensive approach to improve active lifestyles and healthy diet. The activities were tailored to the local school context and delivered through existing school structures (e.g. drawing of the walking trail on the courtyard). The frequency and intensity of the classes were kept moderate to facilitate integration of the classes in curriculum after the intervention. Notwithstanding this, we report effects on muscular strength and speed measured by EUROFIT battery, which was not reported in a previous systematic review [13]. We note that our participants used more time for the speed shuttle run when getting older in both the intervention and control group. This tendency contradicts the current literature that states that (primarily European) adolescents decrease the time on speed shuttle through the transition from 12 to 15 years [43,50]. The explanation of the different findings in Ecuadorian adolescents is speculative and perhaps due to a less favorable environmental conditions for physical activity found in Europe [51], differences in tradition of health promotion programs [15] and genetic factors [52,53].

As observed in the present study, a decline in moderate to vigorous physical activity is common in early adolescence [15]. ACTIVITAL led to a significantly lower decrease in the proportion of adolescents that met the daily recommended 60 min of moderate to vigorous physical activity. Besides, although the intervention effect on the moderate or vigorous activities is borderline significant in the present manuscript, it is relevant as it comprises a quarter of the daily recommendations. Furthermore, this difference was almost three times higher than that reported in a meta-analysis of physical activity interventions in children and adolescents [54] which stated a small improvement on the moderate or vigorous activities of ~4 min/day as measured using accelerometers.

For screen time and sedentary behavior no intervention effects were found. Specific components are needed to address sedentary behavior [55]. We covered screen time in classes only during the first year of the trial, which was possibly insufficient to produce a significant effect.

We found no effects of the intervention on mean BMI or prevalence of overweight. While some authors report favorable effects in girls [56,57] or boys only [58,59], others found no differences [48,49,60]. Only a study from Greece reported a significant effect in both groups after one year [61]. The effect size on these outcomes was possibly too small and our follow-up period was possibly too short to detect effects on BMI.

We observed a large heterogeneity in our intervention effect between the schools. School characteristics could explain the variation on intervention effects among pairs. Speed agility decreased significantly more in pairs with larger size schools. As size of secondary schools is associated with higher academic achievements [62], children from a larger sized school might have a comparative advantage and respond better to the intervention. Educational classes were an important activity in this trial and the uptake of information could have been better in larger schools. Larger sized schools also typically provide more extracurricular activities [62]. In Cuenca, such extra activities are dedicated to sports and physical activity. Vertical jump on the other hand only improved significantly in school pairs that had both male and female students. Physical activity in adolescents is subjected to peer influence [63]. A recent study reported that physical activity of male adolescents was associated with that of their female peers, while female physical activity was associated with physical activity of their male and female peers [64]. We therefore hypothesize that physical activity as well as physical fitness in female adolescents are mainly associated with the presence of males rather than the female peers in schools. Space available for physical activity during recess did not influence the effects of our intervention on physical activity or physical fitness except for the flamingo balance test. This finding is promising for schools with limited space available. We hypothesize that the limited space in school with <4.07 students/m^2^ triggered the adolescents to engage in static games that improve the balance component of fitness [44]. The higher proportion of adolescents with >3 hours of screen time during weekdays in private schools is possibly explained by a higher access to computers at home in adolescents from private schools compared to adolescents from public schools (88% vs. 54% had a computer at home respectively). Also, our results indicate that a higher proportion of adolescents from schools with a half-day class schedule met the recommendations of 60 min physical activity per day compared to those from schools with a full day schedule. Probably, adolescents in schools with a half day schedule had more time to meet the recommendations for time spent on physical activity compared to schools with a full day schedule, as schools with a half day schedule usually have teaching sessions of 45 min with a recess of 35 min while schools with full day schedule have classes of 35 min and a recess of 30 min. However, as the trial was not designed to analyze the moderating effects of the school characteristics on the intervention effects, the explanations for these effects are speculative. In addition, we acknowledge a large variation of the effect in the pairs that was not explained by the recorded school characteristics, which merits further consideration in future trials.

This study has important strengths. First, we delivered a comprehensive intervention aimed at promoting both diet and physical activity. A second strength is the duration of our program, which is longer than most trials on the topic. A third strength is the comprehensive assessment of physical fitness. Most studies evaluate only a few physical fitness components [13]. Finally, the positive findings and feedback from the parents, teachers and adolescents is encouraging and promising to scale up the approach. We think the intervention was successful as it was new and responded to a latent need for activities that address healthy lifestyles. The children and parents valued the practical nature of the recommendations and simplicity of the messages.

A limitation of this study is the large and unbalanced dropout. Frequently changing school is common in Ecuador. Children lost to follow-up were similar to their peers at baseline and the missing data analysis showed no major differences. One school in the control group had an exceptionally high dropout rate (12%) associated with overall very poor academic performance and drug misuse. The assessment of physical activity in a sub-sample is an additional limitation. Although we blinded staff that measured outcomes to the allocation of the schools, we cannot rule out that they observed elements of the interventions such as the posters or the walking trail. We could only assess the use of the walking trail in two schools due to logistical constraints. Although our results are encouraging for school interventions in LMICs, our findings are both mixed and modest. The findings were also not consistent over the outcomes. In addition, the findings for the 20 m shuttle run and bent arm hang should be interpreted with caution since a post-hoc analysis showed a statistical power of 64% and 65% for these outcomes respectively.

The trial included 13% of adolescents between 8^th^ and 9^th^ grade from urban schools in Cuenca that is characterized by mixed *mestizo* ethnicity and its high altitude. Further generalization of our findings is hence limited to urban schools in the regions that share these characteristics [65].

## Conclusion

In conclusion, a comprehensive school-based program to improve diet and physical activity can improve physical fitness in adolescents from urban area of LMICs and can minimize the decline in physical activity levels during early adolescence.

## Abbreviations

BMI: Body mass index
CI: Confidence intervals
LMICs: Low- and middle- income countries
NCDs: Noncommunicable diseases
WHO: World Health Organization
